# A commentary on ‘Meta-analysis of post-transcatheter aortic valve replacement outcomes in patients with cardiac amyloidosis and aortic stenosis’

**DOI:** 10.1097/JS9.0000000000001271

**Published:** 2024-03-11

**Authors:** Mingyue Ji, Juan Pu, Lili Jiang, Xinwei Wang, Caojian Zuo

**Affiliations:** a Department of Cardiology; b Department of Oncology, Lianshui People’s Hospital, Kangda College of Nanjing Medical University, Huai’an, Jiangsu; c Department of Cardiology, Shanghai Deji Hospital, Qingdao University, Shanghai, People’s Republic of China

*Dear Editor,*

It was with great interest that we read the article published in the *International Journal of Surgery*. Jaiswal *et al*.^1^ conducted a meta-analysis of adult patients with cardiac amyloidosis (CA) and aortic stenosis (AS) who underwent post-transcatheter aortic valve replacement (post-TAVR). This meta-analysis is the first to assess clinical outcomes for patients with AS and CA after TAVR. The study’s findings demonstrated that, following TAVR, significant bleeding and short-term mortality were similar in both patient groups. However, compared to patients with AS, the incidence of stroke and acute kidney damage (AKI) was much higher in patients with CA and AS. Nonetheless, we think that this study misses certain important questions.

First, there are noteworthy omissions of essential articles, such as Nitsche *et al*.’s study^2^ on post-TAVR for CA and AS in adults. According to this study, out of 407 patients who were referred for TAVR, 97 (or 24%) passed away after a median of 1.7 years (IQR: 1.3–2.6 years). Major adverse events following TAVR occurred at the same rate in patients with lone AS and AS–CA, according to Valve Academic Research Consortium-2: stroke (2.7% vs. 2.9%), vascular complication (4.7% vs. 2.9%), acute kidney injury (7.5% vs. 6.1%), and pacemaker implantation (6.4% vs. 14.7%) (*P* for all >0.05). All in all, the clinical outcome data following TAVR can be calculated for a meta-analysis. However, this study was just cited and was not included in this meta-analysis, which could have led to bias. In terms of methodology, the inclusion criteria require a more thorough justification.

Secondly, this study does not address the risk of bias or quality assessment. The Risk of Bias in Nonrandomized Studies of Interventions (ROBINS-I) instrument was used by two independent investigators to evaluate the risk of bias in observational studies^3^. Confounding, participant selection, intervention classification, variations from intended interventions, missing data, outcome measurement, and choice of reported results were all evaluated as potential sources of bias.

Lastly, there’s a chance that some lingering confounding factors were missed. In AS, the frequency of CA is constant and rises with age. Male sex, lower BMI, advanced age, and signs of more advanced disease are characteristics of patients with simultaneous CA and AS.

Patients with AS have a worse prognosis when they have both conditions, yet, aortic valve replacement still has major benefits in these cases. Major bleeding and short-term mortality were similar in both patient groups after TAVR, according to the study that was examined; nevertheless, because of the small sample size, more randomized research is needed to fully describe this problem. Additionally, the findings of multiple other meta-analyses point to a number of distinct clinical, electrocardiographic, and echocardiographic characteristics that may serve as ‘red flags’ for CA in individuals with AS^4^. Particularly in the context of TAVR, further research is necessary to determine the best course of action for treating patients with CA and screening for it in older AS patients^5^. For these patients, larger, multicenter prospective studies are desperately needed in order to improve medical decision-making and patient stratification for potential future therapeutic interventions.

## Ethics approval

Not applicable.

## Consent

Not applicable.

## Source of funding

This work was supported by grants from the Shanghai Sailing Program of Talented Youth in Science and Technology (19YF1438800), the National Natural Science Foundation of China (81900414), the Jiangsu Provincial Double-Innovation Doctor Program, and Shanghai Deji Future Talent Program.

## Author contribution

M.J. and J.P.: study concept or design, data collection, data analysis or interpretation, and writing the paper; L.J. and X.W.: data collection; C.Z.: study concept or design, writing, and revising the paper.

## Conflicts of interest disclosure

This manuscript is a comment without conflicts of interest.

## Research registration unique identifying number (UIN)

Not applicable.

## Guarantor

Caojian Zuo.

## Data availability statement

This manuscript is a comment. Don’t need a Data availability statement. However, all the data from the current study are publicly available.

## Provenance and peer review

This manuscript is a comment without being invited.
